# Quantification of Motor Learning in Hand Adjustability Movements: An Evaluation Variable for Discriminant Cognitive Decline

**DOI:** 10.1109/JTEHM.2025.3540203

**Published:** 2025-02-10

**Authors:** Kazuya Toshima, Yu Chokki, Toshiaki Wasaka, Tsukasa Tamaru, Yoshifumi Morita

**Affiliations:** Nagoya Institute of Technology 12982 Aichi 466-8555 Japan; Kaikoukai Rehabilitation Hospital Aichi 490-1405 Japan

**Keywords:** Motor learning, mild cognitive impairment (MCI), quantification, attentional function, cognitive function

## Abstract

Objective: Mild cognitive impairment (MCI) is characterized by early symptoms of attentional decline and may be distinguished through motor learning results. A relationship was reported between dexterous hand movements and cognitive function in older adults. Therefore, this study focuses on motor learning involving dexterous hand movements. As motor learning engages two distinct types of attention, external and internal, we aimed to develop an evaluation method that separates these attentional functions within motor learning. The objective of this study was to develop and verify the effectiveness of this evaluation method. The effectiveness was assessed by comparing two motor learning variables between a normal cognitive (NC) and MCI groups. Method: To evaluate motor learning through dexterous hand movements, we utilized the iWakka device. Two types of visual tracking tasks, repeat and random, were designed to evaluate motor learning from different aspects. The tracking errors in both tasks were quantitatively measured, and the initial and final improvement rates during motor learning were defined as the evaluation variables. The study included 28 MCI participants and 40 NC participants, and the effectiveness of the proposed method was verified by comparing results between the groups. Results: The repeat task revealed a significantly lower learning rate in MCI participants (p <0.01). In contrast, no significant difference was observed between MCI and NC participants in the random task (p =0.67). Conclusion: The evaluation method proposed in this study demonstrated the possibility of obtaining evaluation variables that indicate the characteristics of MCI. Clinical Impact: The methods proposed in this work are clinically relevant because the proposed evaluation system can make evaluation variables for discriminating cognitive decline in MCI. That it, the proposed approach can also be used to provide discrimination for cognitive decline in MCI.

## I. Introduction

Mild cognitive impairment (MCI) is defined as an intermediate stage between age-related cognitive decline and dementia [1], [2]. It is described as a state in which cognitive function is temporarily impaired, and early detection of MCI may prevent progression to dementia [3], [4]. Therefore, developing a method to evaluate cognitive decline is important. MCI is characterized by a mild decline in attention [5], [6], which primarily contributes to the cognitive decline in MCI. Thus, quantification of attentional impairment may be an evaluation variable to discriminate MCI [7]. The Montreal Cognitive Assessment (MoCA) is used to assess mild attentional decline in the context of detecting MCI. MCI is characterized by a decline in selective attention and sustained attention, which reflect overall cognitive function. The MoCA evaluates selective attention using the Digit Span task and sustained attention via the Target Detection task. The outcomes of these tasks provide valuable indicators for distinguishing individuals with MCI [8]. However, attentional function results of the MoCA are influenced by age and education biases [9]. Recent research has revealed a relationship between motor learning results and attentional decline [10], [11]. In addition, the attentional functions involved in motor learning appear to be less biased by individual background factors, making them potentially more accurate indicators of cognitive impairment [12]. Additionally, utilizing equipment to evaluate motor learning has the advantage of mitigating confounding factors associated with the measurement environment [13]. Thus, device-based motor learning evaluations may provide a more reliable method for distinguishing cognitive decline.

Motor learning is the process of improving the accuracy of a target movement through repeated practice. The motor learning results are expressed by quantifying the difference between the target and actual movements [14]. As motor learning requires attentional function, which is reflected in motor learning outcomes, it can be used to assess cognitive function [15]. Especially, motor learning has been thought to involve two types of attentions: external and internal. This difference in attention has been studied by two different research domains using different methods [11], [16]. The external attention in motor learning has been studied in the motor domain. In external attention, which refers to the selection and modulation of sensory information, an external focus facilitates automaticity in motor control and promotes movement efficiency [16]. Internal attention in motor learning has been studied in the cognitive domain. Internal attention refers to directing awareness toward internal information necessary for task execution. This internal information includes task rules (e.g., procedures or conditions for performing actions) and response preparation (e.g., conscious planning for subsequent actions). Internal attention plays a role in selecting, adjusting, and maintaining these pieces of information as needed [17]. Therefore, in efficiently memorizing a movement, internal attention plays a crucial role in recognizing whether the movement is correct. In summary, motor learning involves the interaction of both external and internal attentions [18]. As such, both types of attention may provide valuable evaluation variables for detecting cognitive decline in individuals with MCI.

The method for quantifying attentional functions as external attention is described as follows. A previous study showed the relationship between motor control and external attention using reaching movements with an exoskeleton lever arm like a robot. In this task, participants used the lever arm to manipulate a cursor, aligning it with a target line. The task performance was quantified by measuring the time taken and accuracy with which the cursor reached the target line [19]. To quantify motor learning, combining movements with a task involving visual information is necessary. The previous studies focused on simple and easily comprehendible upper limb reaching movements using an exoskeleton lever arm. In such movements, the mobilization of attentional function required to control and learn the movement may be low [20], [21]. Therefore, selecting tasks that require a higher degree of attention is critical for accurately assessing motor learning and attentional function.

The method for quantifying attentional functions as internal attention is described as follows. To study this function, a paradigm called the serial reaction time task (SRT) has been commonly used [22]. The SRT involves embedding consistent movement patterns into a task, allowing participants to more easily predict movements on recognizing patterns [23], [24]. The result of this pattern recognition is represented in the brain as a feeling of pleasure, which in turn has been shown to facilitate learning [25], [26]. Therefore, internal attention evaluates the process of judging the outcome of a movement based on an internal representation. MCI has been reported to reduce internal attention and in turn reduce motor learning [27], [28]. While motor learning results are associated with cognitive decline, separately quantifying external and internal attention could provide a more precise understanding of MCI characteristics.

Thus, we considered movement selection and task design to quantify external and internal attention through motor learning. To select movements, considering the involvement of attention functions required during exercise was necessary. In particular, as the relationship between attentional function and hand movements has been already established, selecting hand movements is suitable [29]. Regarding the relationship between hand movements and cognitive function, in a study, the difference in brain activity when writing with a pencil versus typing on a tablet was investigated. Activation of the frontal lobe and the orbitofrontal cortex (OFC) has been observed during writing, but there is evidence that brain activity does not appear to increase when characters are entered by simple finger tapping [30]. In other words, the state of fine motor control may promote the mobilization of attentional functions. Therefore, we developed a device called iWakka that uses hand movement through the adjustment process of grip force. Compared with devices commonly used in motor learning studies, iWakka allows objects to be grasped with a small force of <0.5 kg while allowing users to adjust their grip force by referencing the target grip force displayed on the screen. We initiated research using iWakka for the assessment and intervention of stroke patients in 2012 and have been conducting cognitive function assessments continuously since 2019. Furthermore, leveraging its capability to freely design target grip forces, this study introduces repeat and random tasks to facilitate the classification of attentional functions. External attention was assessed by randomly presenting a task line. The changing of the task allows the brain to pay attention to the hand movements. Internal attention can be quantified by motor patterns obtained by repeating the same task [31]. Therefore, a separate evaluation of the two attentional functions is possible. Thus, a task combining the random and repeat tasks was set.

We used iWakka to evaluate the adjustment process of grip force. In addition, amongst the two attentional functions involved in motor learning, attentional decline is an important assessment variable that can differentiate MCI from normal cognitive older adults. Therefore, we developed a task to quantify the two attentional functions separately. This will clearly show the characteristics of attentional decline in MCI. This study was aimed at developing an MCI evaluation method and verifying its effectiveness. The effectiveness was clarified by comparing the two evaluation variables of motor learning between a normal cognitive (NC) and MCI groups.

## II. Materials

### A. Measuring System

**FIGURE 1.**
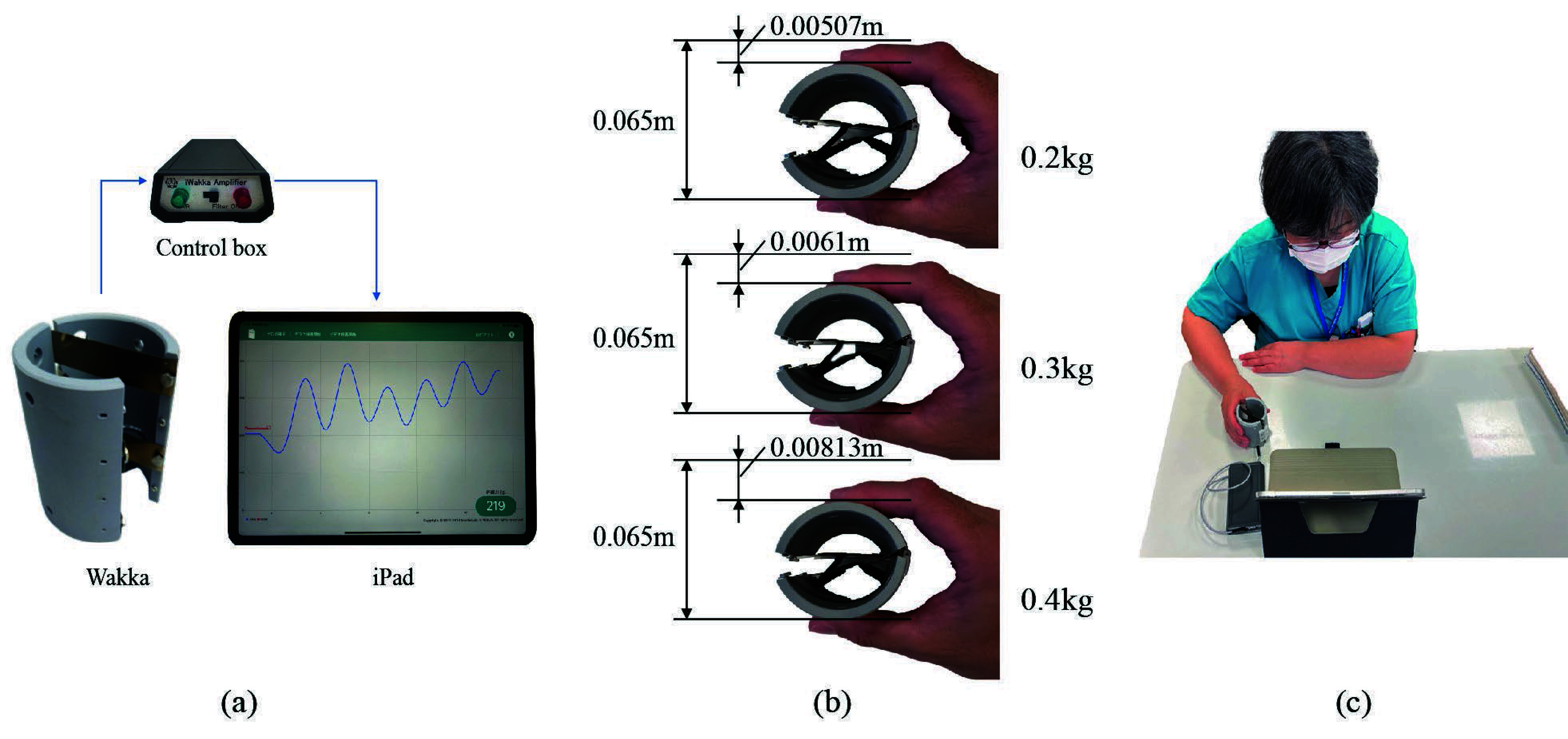
Proposed evaluation system. (a) Overview of the iWakka, (b) Wakka requires a certain amount of force depending on the amount of grip (m), (c)Posture during examination.

Fig. 1 shows the proposed evaluation system. Fig. 1(a) shows the components of the iWakka, namely, a grasping device called the Wakka, a control box, and an iPad (Apple Inc.) [32], [33]. The Wakka has a height of 0.08 m, an outer diameter of 0.065 m, and a weight of 0.112 kg. Its force measurement range is 0–0.5 kg, with a resolution of $1.6\times 10^{-3}$ kg. As shown in Fig. 1 (b), when the Wakka is grasped, a leaf spring inside the Wakka is deformed, resulting in a decrease in its outer diameter. This decrease is defined as the amount of deformation. The relationship between the deformation of the Wakka and the grasping force is linear, with a spring constant of $4.82\times 10^{2}$ N/m, meaning, when a force of $4.92\times 10^{-2}$ kg is applied to the Wakka, it undergoes a deformation of 1 mm. The sampling time for grasping force measurement is 0.1 s.

In the tracking task, one of the evaluation tasks, the user manipulated the Wakka placed on the table to follow the pointer to the target line while adjusting the grasping force. The pointer on the screen indicated the current grasping force, displaying increments and decrements with strong and weak grasps, respectively. In addition, the result of the grasping force, displayed on the monitor, reflected the actual force value; by adjusting the grasping force, the pointer displayed on the screen oscillated up and down, and the user followed the pointer along the target line. Fig. 1(c) shows the posture of the user during the examination.

### B. Evaluation Task Setting

**FIGURE 2.**
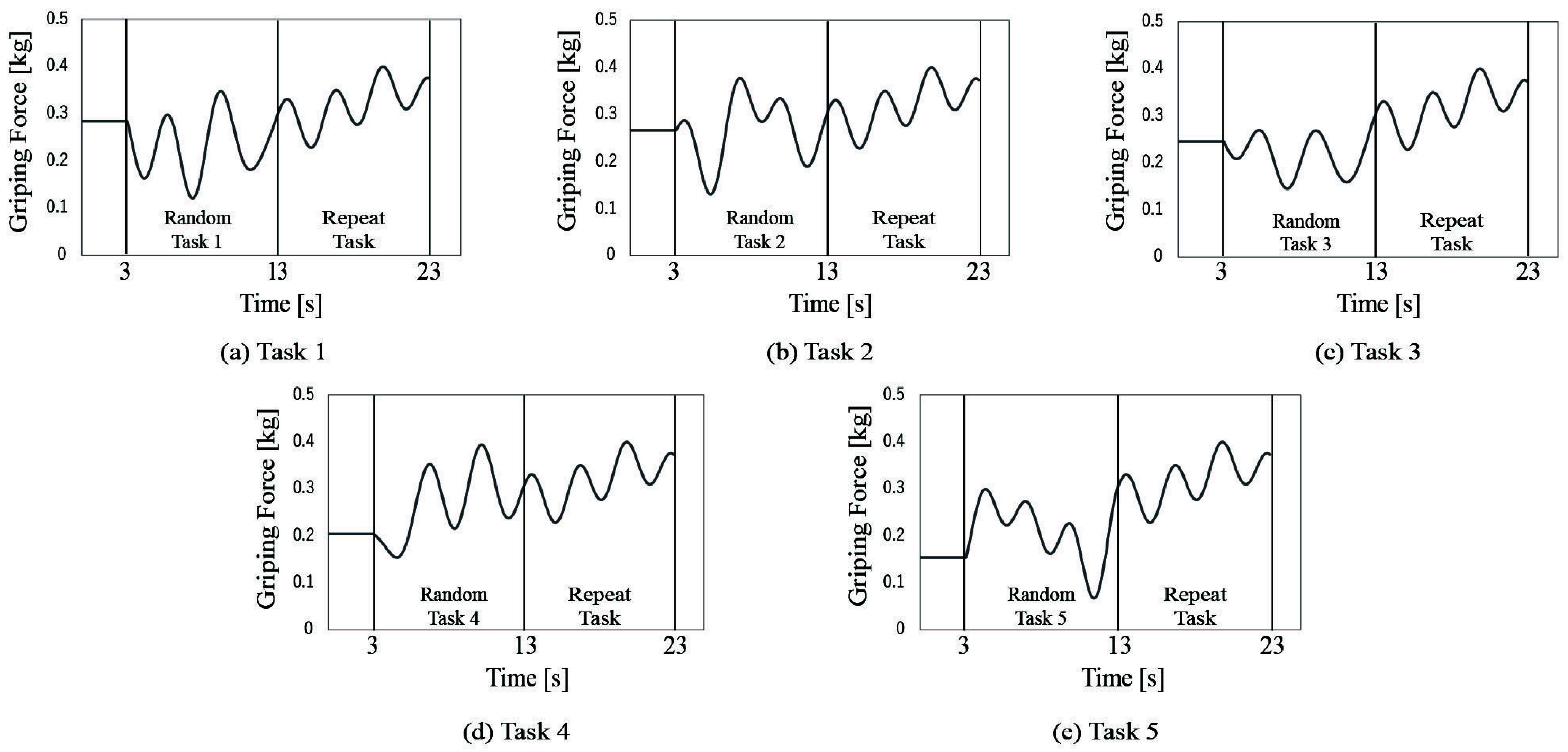
The task is divided into three parts. The first part is a preparation period to align with the task line. The measurement parts are divided into two halves.The first half, from 3 to 13 s, includes a random task. The second half, from 13 to 23 s, includes a repeat task. The first haves include five different tasks,whereas the second halves include only task. Five types of tasks (a)–(e) were set to combine the first and second half.

The random task quantified external attention by analyzing motor adjustments in response to tasks presented in a random order. In contrast, the repeat task quantified internal attention by analyzing motor patterns during repetition of the same task [34]. Therefore, we assessed these two attentional functions separately by designing an evaluation task that combines the random and repetitive tasks. We set up five types of target lines, as indicated in Fig. 2. The target line comprised a constant of 0.25 kg for the first 3 s and a waveform varying from 0.06 to 0.4 kg for the next 20 s. The 20 s target line consisted of two parts: the first (3–13 s) and second (13–23 s) halves of the 20 s target lines were used in random and repeat tasks, respectively. Five different target lines were prepared in the random task. Let *i* denote one of the five different target lines and also the task number. The five types of target lines are formulated as follows:

\begin{align*} f_{d}\left ({{ k }}\right)=\begin{cases} \displaystyle f_{\mathrm {Ran}}^{i}\left ({{ N_{1} }}\right) & (k=1,\cdots,N_{1}) \\ \displaystyle f_{\mathrm {Ran}}^{i}\left ({{ k }}\right) & (k=N_{1}+1,\cdots,N_{1}+N) \\ \displaystyle f_{\mathrm {Rep}}\left ({{ k }}\right) & (k=N_{1}+N+1,\cdots,N_{1}+2N), \end{cases} \tag {1}\end{align*}

where $N_{1}=30$, $N=100$, $f_{\mathrm {Ran}}^{i}\left ({{ k }}\right), (i=1,\cdots,5)$, and $f_{\mathrm {Rep}}(k)$ are the target lines for the random task and the repeat task, respectively, which were defined as;

\begin{align*} f_{\mathrm {Ran}}^{i}\left ({{ k }}\right)& =b_{0}^{i}+\sum \limits _{n=1}^{6} \left ({{ a_{n}^{i}\sin \left ({{ n\omega (k-N_{1})\Delta T }}\right)}}\right. \\ & \quad \left.{{+b_{n}^{i}\cos \left ({{ n\omega (k-N_{1})\Delta T }}\right) }}\right),~\left ({{ i=1,\cdots,5}}\right), \tag {2}\\ f_{\mathrm {Rep}}\left ({{ k }}\right)& =b_{0}+\sum \limits _{n=1}^{6} \left ({{ a_{n}\sin \left ({{ n\omega (k-N_{1}-N)\Delta T }}\right)}}\right. \\ & \quad \left.{{+b_{n}\cos \left ({{ n\omega (k-N_{1}-N)\Delta T }}\right) }}\right), \tag {3}\end{align*}

where $\omega $ is the fundamental frequency and $\mathrm {\Delta }T$ is the sampling time. We set $\omega =2\pi /18$ rad/s and $\mathrm {\Delta }T=0.1$ s. The coefficients of Eqs. (2) and (3) are listed in Table 1. These coefficients, ranging from −5 to 5, were selected to satisfy the following boundary conditions between random and repeat tasks:

\begin{align*} & f_{\mathrm {Ran}}^{i}\left ({{ N_{1}+N }}\right)=f_{\mathrm {Rep}}\left ({{ N_{1}+N }}\right), ~(i=1,\cdots,5) \\ & f_{\mathrm {Ran}}^{i}\left ({{ N_{1}+N-1 }}\right)-f_{\mathrm {Ran}}^{i}\left ({{ N_{1}+N }}\right)\approx f_{\mathrm {Rep}}\left ({{ N_{1}+N }}\right) \\ & \quad -f_{\mathrm {Rep}}\left ({{ N_{1}+N+1 }}\right), ~(i=1,\cdots,5)\end{align*}

**TABLE 1:** Coefficients of Target Lines for Random and Repeat Tasks

$f_{\mathrm {Rep}}(k)$		$f_{\mathrm {Ran}}^{i}(k)$					
		*i*	1	2	3	4	5
$b_{0}$	2.3	$b_{0}^{i}$	-4.2	1.4	1.9	3	-3.9
$a_{1}$	3.4	$a_{1}^{i}$	-1.1	-2.9	-4.6	-0.4	3.4
$a_{2}$	2.5	$a_{2}^{i}$	3.3	-2.2	-0.1	1.8	-2.1
$a_{3}$	-1	$a_{3}^{i}$	3.8	4.6	2.3	3.2	2.7
$a_{4}$	1.1	$a_{4}^{i}$	4	0.6	4	2.1	1.1
$a_{5}$	-2,2	$a_{5}^{i}$	-5	-0.4	-4.3	-2.7	-1.5
$a_{6}$	-2,2	$a_{6}^{i}$	-2.9	4.6	2	-1.9	4.9
$b_{1}$	2.4	$b_{1}^{i}$	-0.2	0.2	-3.8	0.9	-2.7
$b_{2}$	0	$b_{2}^{i}$	-1.1	3.2	2.6	-3.1	-4.5
$b_{3}$	-1.2	$b_{3}^{i}$	-2.2	-4.5	1.4	-1.6	0.6
$b_{4}$	1.8	$b_{4}^{i}$	-5	-0.4	-1.6	4.6	4.8
$b_{5}$	-2.6	$b_{5}^{i}$	-2.6	3.5	3	-2.4	-0.2
$b_{6}$	-3.5	$b_{6}^{i}$	3.8	-2.1	-1.5	0.7	-4.3

### C. Methods for Calculating Evaluation Variable

To evaluate the tracking performance of the repeat and random tasks separately, the mean value of the absolute difference between the target and measured values of the grasping force was calculated for each trial of the repeat and random tasks using the following formula:

\begin{align*} {AGF}_{Ran}=\frac {1}{N}\sum \limits _{k=N_{1}+1}^{N_{1}+N} \left |{{ f_{\mathrm {Ran}}^{i}\left ({{ k }}\right)-f\left ({{ k }}\right) }}\right |, \tag {4}\\ {AGF}_{Rep}=\frac {1}{N}\sum \limits _{k=N_{1}+N+1}^{N_{1}+2N} \left |{{ f_{\mathrm {Rep}}\left ({{ k }}\right)-f\left ({{ k }}\right) }}\right |, \tag {5}\end{align*}

where $f\left ({{ k }}\right)$ is the measured value of the grasping force. The value obtained from Eqs. (4) and (5) is referred to as the adjustability for grasping (AGF) score. A smaller AGF score indicates higher adjustability of grasping force. For evaluation tasks, such as the tracking tasks in this study, considerable variability may exist in the evaluation scores between trials. Moreover, data from individual trials may not provide accurate scores, and the results from multiple trials are recommended to be combined for analysis [35]. Therefore, we examined changes in motor learning using the mean value of AGF scores, referred to as the mean AGF scores, over five trials. A block of every five trials was considered, starting from the first trial. For example, the mean AGF scores of the *j*-block for the repeat and random tasks are denoted as $\overline {AGF}_{\mathrm {Ran}}^{j}$ and $\overline {AGF}_{\mathrm {Rep}}^{j}(j=1,2,\cdots,10)$, respectively.

To separately evaluate the results of external and internal attentions in motor learning, the learning rates *LR_Rep_* and *LR_Ran_* were calculated using the mean AGF scores of the first and tenth blocks as

\begin{align*} {LR}_{\mathrm {Rep}} & =\frac {\overline {AGF}_{\mathrm {Rep}}^{1}-\overline {AGF}_{\mathrm {Rep}}^{10}}{\overline {AGF}_{\mathrm {Rep}}^{10}}\times 100 \left [{{ \% }}\right ] \tag {6}\\ {LR}_{\mathrm {Ran}} & =\frac {\overline {AGF}_{\mathrm {Ran}}^{1}-\overline {AGF}_{\mathrm {Ran}}^{10}}{\overline {AGF}_{\mathrm {Ran}}^{10}}\times 100 \left [{{ \% }}\right ] \tag {7}\end{align*}

Positive values of the motor learning rate indicated a learning effect, and larger positive values indicated a higher learning effect.

## III. Methodology

### A. Experimental Participants

We recruited 68 community-dwelling older adults. The inclusion criteria for participants were as follows: (1) community-dwelling individuals aged 60 years or older, (2) no known history of stroke or neurological disorders that could affect cognitive function, and (3) no known history of peripheral neuropathy or orthopedic disorders that could impair motor function. Then, the participants were categorized into two groups: NC and MCI. In accordance with previous research, NC and MCI were discriminated through comprehensive neuropsychological evaluations using the Mini-Mental State Examination (MMSE) and the Clinical Dementia Rating (CDR), respectively. NC was defined as individuals with MMSE score $\ge 23$ and a CDR score =0, and MCI was defined as individuals with a CDR score =0.5 or 1.0 regardless of the MMSE score [36]. Informed consent was obtained from all the participants before the study was conducted. This study was performed with the ethical approval of the Nagoya Kyoritsu Hospital (k-140) and Nagoya Institute of Technology (2021-15).

The characteristics of each group are listed in Table 2. The group characteristics were confirmed by the results of each cognitive function test. The MMSE was used as a general cognitive function test, the FAB was used to evaluate frontal lobe function, the Trail Making Test partA/partB (TMT-A/-B) was used to evaluate attention function, and the Rey-Osterrieth Complex Figure Test copy/recall 3 min (ROCFT copy/recall 3 min) was used to evaluate memory function. In addition, since mental status also affects cognitive function, the Beck Depression Inventory-II (BDI-II) was used as a self-report measure of depression.

**TABLE 2:** Demographic and Clinical Characteristics of the Participants

Measure	MCI (N =28)	NC (N =40)	95 CI for mean Difference		P-value
	mean (SD)	mean (SD)	lower	upper	
Age [years]	80.75 (6.3)	75.05 (5.9)	2.6	8.7	$\ast $
Sex					
Male	4	10			
Female	24	30			
Education [years]	10.7 (2.1)	10.6 (1.8)	-1	0.8	n.s
General cognitive state					
MMSE	26.9 (1.5)	29.1 (2.6)	-3.2	-1	$\ast \ast \ast $
FAB	12.7 (1.7)	15.6 (3.3)	-4.3	-1.6	$\ast \ast \ast $
TMT-A [s]	66.1 (16.2)	49.6 (37.1)	1.3	31.6	n.s
TMT-B [s]	205.4 (41.5)	122.8 (110.7)	38	127.2	$\ast \ast \ast $
ROCFT-copy	33.3 (2.2)	34.3 (2.9)	-2.4	0.2	n.s
ROCFT-recall [3 min]	12.3 (5.9)	14.6 (5.1)	-8.2	-2.9	$\ast \ast \ast $
BDI-U	1.4 (1.7)	1.1 (1.7)	-0.5	1.1	n.s

Data are expressed as means (standard deviation). Abbreviations: MMSE (Mini Mental State Examination), FAB(Frontal Assessment Battery), TMT-A/-B (Trail Making Test part A/B), ROCFT(Rey-Osterrieth Complex Figure Test copy/recall(3 min)), BDI-II(Beck Depression Inventory-Second Edition). * and ***indicate p <.001 and p<0.05, respectively; n.s indicates not significant.

### B. Experimental Procedure

The participants were instructed about the iWakka, ways to use the iWakka, and the tracking task. They were instructed to keep their eyes on the target line and focus on the error between the target line and actual grasping force displayed on the screen. They operated the Wakka with their dominant hand.

**FIGURE 3.**
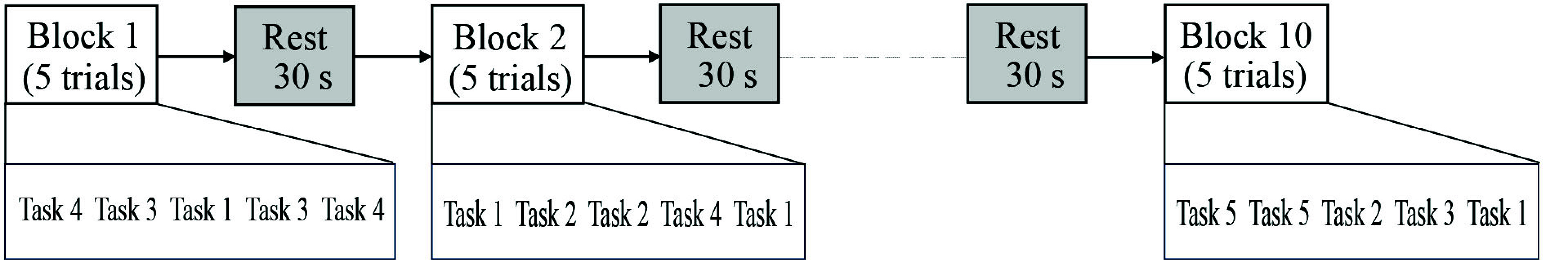
Experimental protocol.

The experimental protocol is shown in Fig. 3. Participants practiced for 3 min beforehand. The tracking tasks were randomly presented as task numbers 43134, 12241, 51544,...from the five tasks shown in Fig. 2. The task was repeated five times in a row as a block. A rest period of 30 s was allowed between blocks. Thus, the total trial time was 19 min and 10 s (23 s $\times 5$ repetitions $\times 10$ blocks). The total duration of the experiment was approximately 30 min including device operation instructions, trial time, and rests. The order of the tasks was the same for all participants.

### C. Statistical Analysis

All statistical analyses were performed using SPSS version 28 (IBM Corporation, Armonk, NY, USA), in this study. The Shapiro-Wilk test was performed to confirm normality and select an appropriate statistical method. Differentiation between the MCI and NC groups was accomplished using a paired t-test for age and education and Mann-Whitney’s U test for MMSE, FAB, TMT-A/-B, ROCFT-copy, ROCFT-recall (3 min), and BDI-II. To evaluate the time-course data across the ten blocks between groups, the mean AGF scores for the random and repeat tasks in both the NC and MCI groups were analyzed using repeated measures analysis of variance (RM-ANOVA). The RM-ANOVA was conducted with the assumption of sphericity tested using Mauchly’s test. On the basis of the results, the Greenhouse-Geisser correction was applied. Time-course (i.e., the number of ten blocks) and task type (i.e., random and repeat tasks) were treated as within-subject factors, while group (i.e., NC and MCI) was treated as a between-subject factor. If significant interactions were observed in the RM-ANOVA, the learning rates represented by *LR_Ran_* and *LR_Rep_* could be important variables for discriminating between NC and MCI. Consequently, a Mann-Whitney U test was used for between-groups, while a Wilcoxon signed-rank test was used for within-group comparisons of *LR_Ran_* and *LR_Rep_*.

## IV. Results

### A. Characteristics Between Groups

Following observations can be drawn from Table 1: 1) The age of the MCI group was significantly higher than that of the NC group. 2) In particular, the MCI group had significantly lower scores than the NC group in terms of the MMSE, FAB, TMT-B, and ROCFT-recall (3 min).

### B. Time Course of Motor Learning

**FIGURE 4.**
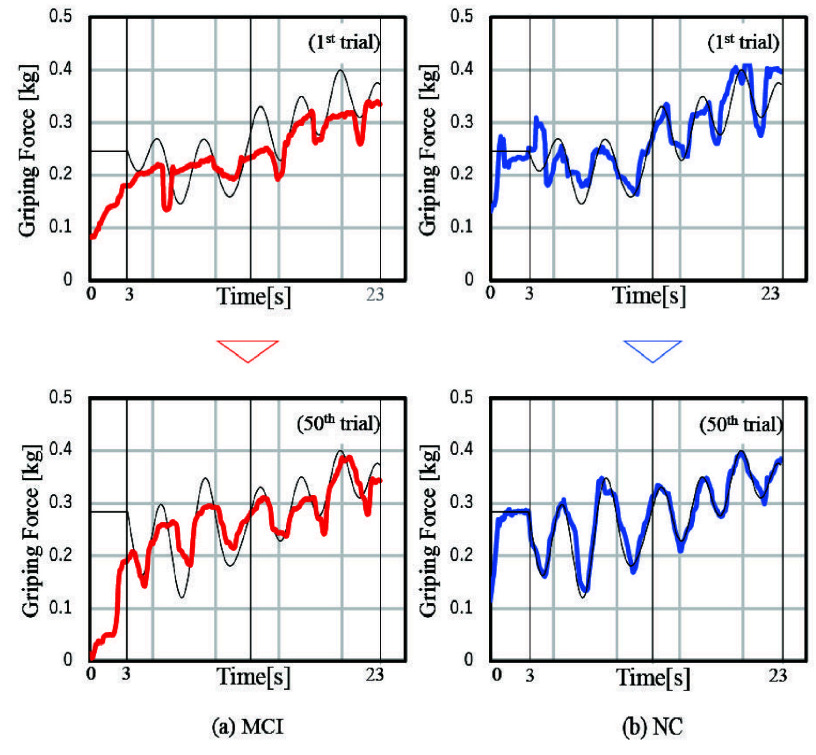
The results on the 1st and 50th trials for individual with MCI and NC.

**FIGURE 5.**
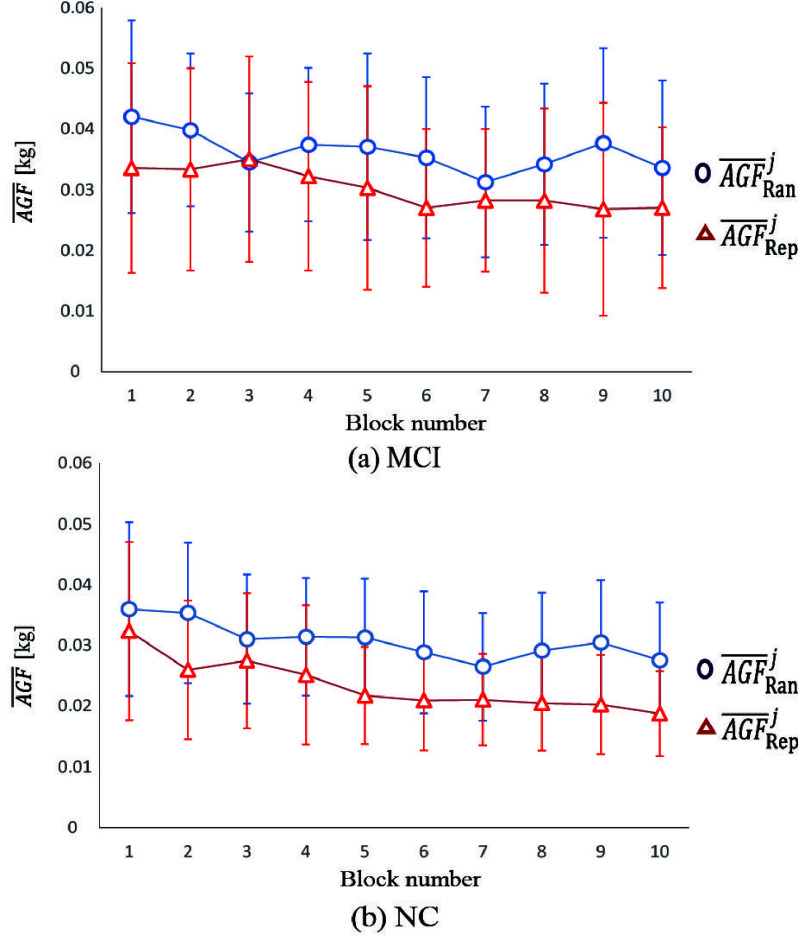
Show results time course. The mean values of the AGF of the jblockfor the repeat and random tasks are denoted $\overline {AGF}_{\mathrm {Ran}}^{j}-\overline {AGF}_{\mathrm {Rep}}^{j} (j=1,2,\cdots,10)$.

Fig. 4 shows the evaluation task results from the 1st and 50th trials for representative MCI and NC participants, suggesting differences in motor learning between the MCI and NC groups. Figs. 5a and 5b illustrate the time course of mean AGF scores across the random and repeat tasks for both the MCI and NC groups. The AGF scores in the NC group gradually decreased, indicating progress in motor learning. In contrast, the AGF score reduction in the MCI group exhibited a different pattern compared to the NC group, particularly in repetitive tasks. These findings are supported by the results of the RM-ANOVA analysis presented in Table 3. The analysis revealed significant first-order interactions between time course and task, indicating a difference in motor learning outcomes between the random and repeat tasks. Additionally, significant second-order interactions were observed, suggesting different time courses of motor learning between MCI and NC groups depending on the task.

**TABLE 3:** Analysis Results of RM-ANOVA

Datasets	F	df	P
Time course	28.3	4.7	$\ast \ast $
Tasks	80.2	1.0	$\ast \ast $
Groups	5.5	1.0	$\ast $
Effect modification			
Time course $\times $ Tasks	8.6	6,1	$\ast \ast $
Tasks $\times $ groups	0.5	1.0	n.s.
Time course $\times $ groups	0.8	4.7	n.s.
Time course $\times $ Tasks $\times $ groups	2.1	6.1	$\ast $

Note: Time course: 1 to 10 blocks, Tasks: Random and repeat tasks, Groups: MCI and NC groups. *, **, and n.s. indicate p<.001, p<0.05, and not significant, respectively

### C. Evaluation Variable for Motor Learning

**FIGURE 6.**
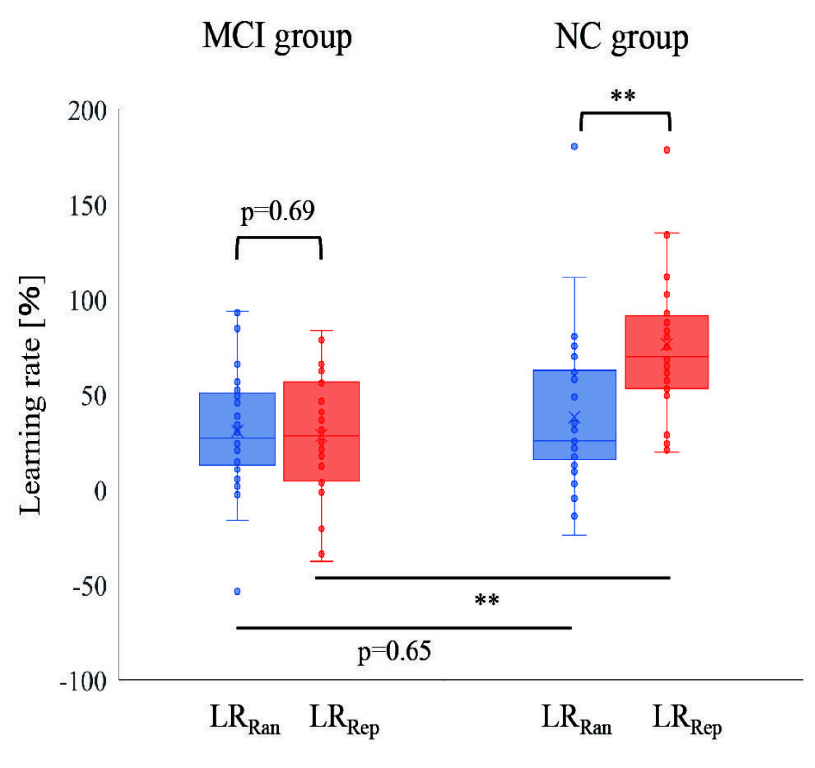
The difference between 1 block and 10 blocks was expressedas a percentage. The results of Repeat task was expressed as $LR_{Rep}$. Theresult of Random task was expressed as $LR_{Ran}$. $LR_{Rep}$ and $LR_{Ran}$ werecompared within the MCI and NC. $LR_{Rep}$ and $LR_{Ran}$ were thencompared within each group. Indicates, $^{\ast \ast }$ p¡0.01, n.s.: not significant.

Fig. 6 shows the motor learning rates, *LR_Rep_* and *LR_Ran_*, for the random and repeat tasks, respectively, in both the MCI and NC groups. In the MCI group, the *LR_Ran_* was 30.8% $\pm ~32.4$% (mean ± SD), and the *LR_Rep_* was 28.5% $\pm ~32.5$%. In the NC group, the *LR_Ran_* was 34.6% $\pm ~36.9$%, and the *LR_Rep_* was 71.8% $\pm ~47.4$%. First, the results were compared between groups. The *LR_Rep_*, which reflects motor learning related to internal attention, was significantly lower in the MCI group (95% credible interval [CI]: −62.7% to −23.9%). In contrast, *LR_Ran_*, which reflects motor learning related to external attention, exhibited no significant difference between the groups (95% CI: −20.7% to 13.1%). These findings indicated a reduction in *LR_Rep_* values for the MCI group compared with the NC group. Next, we compared the results within each group. No significant difference was observed between *LR_Rep_* and *LR_Ran_* in the MCI group (95% CI: −9.5% to 14.1%). However, a significant difference was observed between *LR_Rep_* and *LR_Ran_* in the NC group (95% CI: −51.3% to −23%). These results suggest that the specific decrease in *LR_Rep_* in the MCI group may serve as an evaluation variable indicating the presence of MCI.

## V. Discussion

### A. Differences Between Groups of Characteristics

The characteristics of the groups are listed in Table 2. This study was aimed at developing an evaluation method and verifying its effectiveness. The effectiveness of MCI evaluation method was confirmed by the comparison of the measured values between NC and MCI. First, differences between the groups were analyzed. Memory function (ROCF-recall (3 min)) and attention function (TMTB and FAB) scores were significantly lower in the MCI group. Additionally, age was significantly higher in the MCI group. Dementia can be determined using several criteria, including an MMSE score of $\le 23$, an FAB score of $\le 12$, a TMT-A completion time of $\ge 98.5$ s, a TMT-B completion time of $\ge 188.5$ s, and an ROCFT-recall (3 min) score of $\le 10$ [37], [38], [39], [40], [41]. The MCI group had assessment scores within the normal range, indicating they did not meet the criteria for dementia. However, the MCI group showed impairments in attention and memory compared to the NC group, which aligns with the characteristics of MCI reported in previous studies [5], [6]. Next, we considered the age difference because motor learning has been shown to decline with age. According to a previous study, this decline is due to a decrease in sensory sensitivity as a result of age-related changes [42], [43]. However, we interpreted the effect of age differences to be small in this study for two reasons: 1) the previous study reported that the decline of sensory organs becomes gradual after a certain age [44]. In this study, the NC group was 75 years old and the MCI group was 80 years old, both of which were elderly. 2) the previous study showed that although the older adults experience a decline in motor accuracy, their motor adaptation remains intact [45]. However, older adults tend to exhibit increased vascular comorbidity and chronic disease with aging [46]. These conditions can influence motor learning. In this study, there were limitations in thoroughly verifying comorbidities among the participants. Therefore, it is necessary to recognize the potential impact of age differences on learning as a limitation of the study.

### B. Repeat Task Results for Internal Attention

Two attentional functions were quantified through motor learning. These results are presented in Fig. 6. A within-group comparison of *LR_Ran_* and *LR_Rep_* revealed no significant differences in the MCI group. In contrast, a significant tendency for higher *LR_Rep_* values was observed in the NC group. This finding indicated that the learning effect of *LR_Rep_* was not enhanced in the MCI group. Furthermore, between-group comparisons revealed that the *LR_Rep_* values were significantly lower in the MCI group. Specifically, the mean difference in *LR_Rep_* between the NC and MCI groups was 43.3%, with a 95% CI ranging from −62.7% to −23.9%. This CI suggests that *LR_Rep_* was 23.9%–62.7% lower in the MCI group compared with the NC group. These findings indicate that *LR_Rep_* decreased specifically in the MCI group and is a useful indicator for the diagnosis and evaluation of MCI. Based on these findings, we considered the relationship between the pathology of MCI and internal attention required for motor learning. MCI is characterized by impaired attentional function [5], [6]. Moreover, decline in attentional function in MCI is reflected in behavioral changes before in cognitive decline [47]. These behavioral changes include reduced social activities and decreased motivation, both of which have been associated with reduced dopamine activation [28]. This dopamine activation is associated with internal attention. We considered that reduced internal attention due to reduced dopamine activation was one of the factors that reduced motor learning. The presence of movement patterns improves the match between prediction and movement. The presence of consistent movement patterns enhances the match between predicted and actual movements, leading to a sense of pleasure. This sensation is perceived as a reward, triggering dopamine activation and further facilitating motor learning. Therefore, the lower *LR_Rep_* observed in the MCI group compared to the NC group suggests a reduction in internal attention in MCI, which could serve as a potential evaluation variable for identifying cognitive decline. This is also supported by research on brain function. Previous studies have reported impairments in the OFC, a brain area that is activated when paying attention to the results of one’s own movements to recognize motor patterns [24], [48]. Additionally, bidirectional connections between the hippocampus and OFC have been confirmed [49]. OFC dysfunction has been observed in both dementia and MCI, leading to impairments in attending to the results of internal representations. The decrease in *LR_Rep_* reflects a reduction in attention to internal representations, supporting its potential as a useful evaluation variable for distinguishing MCI.

### C. Results of Random Task for External Attention

The *LR_Ran_* obtained from the random task was compared between the NC and MCI groups, but no significant difference was found. *LR_Ran_* reflects external attention during motor control. When the attentional function in MCI is impaired, the *LR_Ran_* is predicted to decrease. However, no significant difference was observed in this study. The reason for this was considered. A researcher group investigated brain activity during motor control [50]. According to them, attention function in motor control is related to the frontal lobe [50]. The previous studies have shown that this attentional function is the selection and maintenance of attention and is necessary for motor control. While many studies have demonstrated a decline in frontal lobe function in older adults, motor learning has also been shown not to significantly decline with age. This has been attributed to compensatory mechanisms in the brain that help sustain motor learning. For instance, researchers have investigated changes in motor control associated with aging by comparing brain activity patterns during motor learning between young and older adults via neuroimaging. While young adults exhibited prominent activity primarily in motor-related regions, such as the primary motor cortex and supplementary motor area, older adults exhibited increased activity in not only these regions but also additional brain areas, including the prefrontal cortex and parietal lobes [51]. Additionally, older adults have been shown to exhibit higher activation rates in the parietal and occipital lobes compared to younger individuals, compensating for frontal lobe function [52], [53]. This compensatory effect has also been observed in individuals with MCI [6]. Therefore, the absence of significant differences in *LR_Ran_* between the MCI and NC groups may be due to the maintenance of external attention through these compensatory mechanisms in the brain.

### D. Limitation and Future Work

Finally, we considered the use of the two evaluation variables for our future study owing to the relationship between the two evaluation variables and cognitive function. In other words, the two evaluation variables are related to cognitive function from different perspectives. Internal attention may decline before a decline is observed in general cognitive tests. Therefore, we believe that *LR_Rep_* is effective in detecting MCI at an earlier stage. Conversely, external attention is maintained by the compensatory action of the brain. However, the extent of compensation has limitations, and beyond a certain situation, *LR_Ran_*, which is external attention, may also show a decline. Therefore, constructing a discriminant model that adds two discriminant variables may not only be able to identify MCI, but also indicate its severity. Therefore, in our future study, we aim to target challenge of constructing a discriminant model by increasing the number of participants with different cognitive functions. Furthermore, it is essential to advance research on motor learning in not only individuals with MCI but also those with dementia. Such investigations can clarify the progression of motor learning from MCI to dementia. This is expected to enhance our understanding of the severity of MCI and increase the accuracy of clinical assessments.

## VI. Conclusion

In this study, we quantified motor learning via the proposed task involving hand movement with an adjustment process of gripping force. The proposed evaluation tasks could evaluate a difference in cognitive function. The motor learning results of this study showed differences between NC and MCI. In particular, a significant difference was observed between NC and MCI in the *LR_Rep_* obtained from the repeat task. This was thought to indicate a characteristic of MCI and suggests that it may be possible to use it as an evaluation variable to determine MCI.
